# A rapid assessment of the implementation of integrated disease surveillance and response system in Northeast Nigeria, 2017

**DOI:** 10.1186/s12889-020-08707-4

**Published:** 2020-05-01

**Authors:** Luka Mangveep Ibrahim, Mary Stephen, Ifeanyi Okudo, Samuel Mutbam Kitgakka, Ibrahim Njida Mamadu, Isha Fatma Njai, Saliu Oladele, Sadiq Garba, Olubunmi Ojo, Chikwe Ihekweazu, Clement Lugala Peter Lasuba, Ali Ahmed Yahaya, Peter Nsubuga, Wondimagegnehu Alemu

**Affiliations:** 1 World health Organization, Rivers House, #83 Ralph Shodeinde Street, Abuja, Nigeria; 2 WHO Africa Regional Office, Brazzaville, Congo; 3 Nigerian Center for Disease Control, Jabi, Abuja Nigeria; 4 Global Public Health Solutions, Atlanta, GA USA; 5 International Health Consultancy, Atlanta, GA USA

**Keywords:** Integrated disease surveillance and response, Implementation, Rapid assessment, Nigeria

## Abstract

**Background:**

Integrated disease surveillance and response (IDSR) is the strategy adopted for public health surveillance in Nigeria. IDSR has been operational in Nigeria since 2001 but the functionality varies from state to state. The outbreaks of cerebrospinal meningitis and cholera in 2017 indicated weakness in the functionality of the system. A rapid assessment of the IDSR was conducted in three northeastern states to identify and address gaps to strengthen the system.

**Method:**

The survey was conducted at the state and local government areas using standard IDSR assessment tools which were adapted to the Nigerian context. Checklists were used to extract data from reports and records on resources and tools for implementation of IDSR. Questionnaires were used to interview respondents on their capacities to implement IDSR. Quantitative data were entered into an MS Excel spreadsheet, analysed and presented in proportions. Qualitative data were summarised and reported by thematic area.

**Results:**

A total of 34 respondents participated in the rapid survey from six health facilities and six local government areas (LGAs). Of the 2598 health facilities in the three states, only 606 (23%) were involved in reporting IDSR. The standard case definitions were available in all state and LGA offices and health facilities visited. Only 41 (63%) and 31 (47.7%) of the LGAs in the three states had rapid response teams and epidemic preparedness and response committees respectively. The Disease Surveillance and Notification Officers (DSNOs) and clinicians’ knowledge were limited to only timeliness and completeness among over 10 core indicators for IDSR. Review of the facility registers revealed many missing variables; the commonly missed variables were patients’ age, sex, diagnosis and laboratory results.

**Conclusions:**

The major gaps were poor documentation of patients’ data in the facility registers, inadequate reporting tools, limited participation of health facilities in IDSR and limited capacities of personnel to identify, report IDSR priority diseases, analyze and interpret IDSR data for decision making. Training of surveillance focal persons, provision of IDSR reporting tools and effective supportive supervisions will strengthen the system in the country.

## Background

Increasing globalization has meant increasing vulnerability for countries and communities to the potential threat of emerging infectious disease outbreaks and pandemics across the globe [1]. To safeguard against these global threats, the International Health Regulations (IHR 2005) provide the platform for early notification of all events that might constitute public health emergency of international concern. These regulations are strengthened by domestic public health surveillance of a country [2, 3]. Therefore, the health of a nation is directly related to the effectiveness and operational quality of its public health surveillance system [4]. A strong public health surveillance system requires the availability of the right information for public health action. This information provides the foundational knowledge and scientific information essential for informed decision making and guides appropriate public health planning and interventions including responses to outbreaks of diseases and events [5–8].

In 2001, Nigeria adopted Integrated Disease Surveillance and Response (IDSR) as the strategy for strengthening national public health surveillance and response. IDSR is a framework implemented to improve the usability of surveillance and laboratory data, and improve detection and response to the primary causes of morbidity and mortality in African countries. Its key objectives were 1) to enable early detection and immediate response to acute public health concerns and 2) to assess specific public health problems with a focus on long-term trends and epidemiological patterns to identify the impact of diseases in the country. IDSR also guides, monitors and assesses the impact of interventions; provides a framework for identifying major public health problems in a community; and serves as a planning guide.

In Nigeria, IDSR operates at all levels of the administration (local government area, state and the federal). The national technical guidelines define the specific roles and responsibilities for each level. According to the technical guidelines, the following three categories of priority diseases and conditions are under surveillance.

1. epidemic-prone diseases including those that are notifiable under IHR 2005
2. diseases targeted for elimination and eradication
3. other diseases and conditions of public health importance.

The IDSR requires immediate notification of suspected outbreaks, weekly reporting of all the epidemic-prone diseases and those targeted for elimination and eradication including “zero” reporting and monthly reporting of all other priority diseases and conditions under surveillance.

Health facilities are the basic operational units in the Local Government Area (LGA) and surveillance data is sourced from health facility registers. A designated focal point in each health facility enters data from the patient medical record in the facility register while the surveillance focal person extracts the data from the health facility register into the IDSR reporting forms. The LGA Disease Surveillance and Notification Officers (LGA DSNOs) provide technical support to the health facility surveillance focal persons on: event detection, verification, reporting, sample collection/transportation and responding to outbreaks. They also provide support supervision for the health facility surveillance focal persons. The LGA DSNOs are responsible for collating the health facility reports within the LGA into an LGA summary form and submit to the state health commission.

At the state level, the State DSNOs and the state epidemiologists collate the reports from all LGAs into a state summary form and submit to the Nigeria Center for Disease Control (NCDC) at the national level. The national guidelines specify time schedule for the transmission of the IDSR forms from the lowest operational units to the national level. The deadlines are used to calculate timeliness of reporting across the levels of the surveillance system as timeliness of reporting is one of the core performance indicators for the national surveillance system [9].

The local IDSR reporting system from health facility to LGA is paper-based and is dependent on manually summarizing and transmission of the reports. Implementation of IDSR involves a complex interaction of actors and failure of one or more actors will affect the quality, reliability, completeness, and timeliness of the data and reporting, and in turn impact the overall decision making pathway and public health actions. Recent outbreaks such as the outbreak of cerebrospinal meningitis that caused over 1000 deaths and the 2017 cholera outbreak revealed problems with the functionality of the IDSR system in Nigeria. The weak surveillance system was worsened by the current humanitarian crisis in the north-east of the country, especially Adamawa, Borno and Yobe states.

Disease surveillance is one of the critical components of response to any humanitarian crisis due to the high risk of disease outbreaks as they are a major cause of excess deaths in humanitarian crises [10–12]. Limiting the morbidity and mortality caused by disease outbreak depends on a functional disease surveillance system with rapid detection and response [13]. With the humanitarian crisis and a weak disease surveillance system identified as major risk factors, a rapid assessment was commissioned to determine the status of implementation of IDSR in Adamawa, Borno and Yobe states in North-East Nigeria. The assessment also seeks to collect best practices and identify areas for improvement to strengthen the IDSR implementation in the states.

## Methods

### Study design

A cross-sectional survey was utilized to assess the implementation status of the IDSR strategy at the state, local government area and health facility levels in Adamawa, Borno and Yobe states in North East Nigeria. Respondents at the state level were state epidemiologists, State Disease Surveillance and Notification Officers (SDSNOs) and their assistants, and World Health Organization (WHO) cluster consultants. Respondents at the LGA level were the LGA DSNOs and WHO LGA facilitators and at the health facility level were the health facility surveillance focal persons and head clinicians (the officer in charge of the health facility).

### Survey setting

Nigeria operates a federal system of government made up of 36 states and a Federal Capital Territory (FCT) with 774 constitutionally recognized LGAs grouped into six geopolitical zones: North Central, North East, North West, South East, South South, and South West. Our study area, the North East geopolitical zone, has six states, Bauchi, Taraba, Gombe, Adamawa, Yobe and Borno, with a combined estimated population of 26 million people [14] has been in a humanitarian crisis since 2009. The survey was conducted in the three states (Adamawa, Borno and Yobe), worst affected by the on-going humanitarian crisis. In total, these states have 65 LGAs (Adamawa-21, Borno-27, and Yobe-17) and a combined 2598 health facilities. Two LGAs were selected from each state for the study with a focus on LGAs within or close to the state capitals. In each selected LGA, the list of all health facilities were reviewed with the DSNO and one health facility closest to the LGA headquarters was selected. LGAs and health facilities within or close to the state capital were selected on the premise that these were areas where the system was most likely to still be functional.

### Data collection technique

The data collection tools (checklist and questionnaires) were adapted from the WHO Regional Office for Africa (WHO-AFRO) assessment protocol for national disease surveillance systems and epidemic preparedness and response [15]. The checklists were developed to extract data from documents at the state, LGA and health facility level. The information collected including number and types (public or private) of health facilities participating in IDSR; human and materials resources for implementation of the IDSR strategy including tools for reporting IDSR data; laboratory supports for outbreak investigation and response; past outbreak reports; reports of supervisory visits; and completeness of patient data from the health facility registers. Semi-structured interviewer administered questionnaires were used for a face-to-face interview with the respondents at all levels of reporting based on the required functions at each level according to the IDSR technical guideline. The respondents at the state level were interviewed on concepts and the core indicators of IDSR strategy; standard case definitions of the priority diseases, conditions and events for reporting under IDSR; data management; and support structures available for IDSR implementation, supervision, monitoring and feedbacks. At the LGA level, respondents were interviewed on detection, registration, and reporting of priority diseases; analysis of IDSR data; supervision and feedback to the lower levels; and epidemic preparedness and response to outbreaks. At the health facility level, the questionnaire focused on standard case definitions of priority diseases; core IDSR indicators at the health facility level; extraction of data from health facility registers into the IDSR reporting forms; and completing the IDSR reporting forms. A team of four persons conducted the face-to-face interviews with all the respondents.

### Data analysis

Data were entered into an MS Excel spreadsheet, analysed and presented as proportions and standpoints summaries.

### Ethical considerations

We obtained ethical clearance for the survey from the National Health Research Ethics Committee of Nigeria (NHREC) in the Department of Planning Research and Statistic of the Federal Ministry of Health Nigeria. Verbal informed consent was also obtained from all respondents involved in the study.

## Results

A total of 34 respondents participated in the rapid survey from six LGAs and six health facilities in the three states. Respondents included three state epidemiologists, six state DSNOs and assistant SDSNOs, five LGA DSNOs, six health facility surveillance focal persons, six clinicians and eight WHO cluster consultants and WHO LGA technical facilitators. Of the 65 LGAs in the three states surveyed 63 (96.9%) had DSNOs. Furthermore, only 606 (23%) of the 2598 health facilities in the three states were involved in reporting IDSR in the last twelve months. Of the 127 private health facilities, only 5 (3.3%) were involved in reporting IDSR in the last twelve months before the survey (Table 1) and all the health facilities reporting IDSR were focal sites for surveillance of acute flaccid paralysis (AFP).

**Table 1:** Distribution of Local Government Areas with Rapid Response Teams and facilities reporting IDSR by states in North eastern Nigeria, 2017

States	Population × 1,000,000	Number of Local Government Areas	Local Government Areas with Rapid Response Team	Local Government Areas reporting IDSR	Total functional Health facilities	Health facilities reporting IDSR	Total functional private health facilities	Private health facilities reporting IDSR
Adamawa	3.2	21	14 (66.7%)	21(100%)	793	140 (17.7%)	80	2(2.5%)
Borno	4.2	27	22 (85.2%)	25 (93%)	489	339 (69.3%)	29	2(6.9%)
Yobe	2.3	17	5 (29.4%)	17 (100%)	516	127 (24.6%)	18	1(5.6%)
**Total**	**9.7**	**65**	**41(63.1%)**	**63(96.9%)**	**2598**	**606(23.3%)**	**127(4.9%)**	**5(3.3%)**

Review of the health facility registers revealed that more than one type of registers was in use in the states and some of the patients’ data such as age, sex, diagnosis and laboratory results were missing. Documentation on laboratory investigations was limited to the rapid diagnostic test (RDT) for malaria and not for other diseases requiring laboratory confirmation. All states and LGAs surveyed had the list of standard case definitions and the national IDSR technical guidelines, but there was no evidence of usage of the guidelines. Motorbikes for surveillance activities were not available in the state and LGA offices and only 54.5% of the state epidemiologists, state and LGA DSNOs had computers to support surveillance activities. In the state and LGA offices surveyed, analysis of IDSR data was limited to polio eradication initiative activities and therefore did not focus on other diseases. Only 31 (47.7%) of the LGAs had epidemic management committees, most of them were not functional. Rapid response teams were present in 41 (63%) of the LGAs but only become functional when there were outbreaks of diseases, conditions or events. Only 6 (54.5%) of the 11 respondents noted access to computers. All respondents noted the availability of both IDSR technical guidelines and list of standard case definitions.

The monthly reporting forms were available in only four (67%) of the six health facilities visited. Errors were observed in about two third of the completed monthly IDSR forms. Knowledge of respondents on standard case definitions of priority diseases were limited to the epidemic prone diseases. Knowledge of core indicators for IDSR were limited to timeliness and completeness of IDSR reports. The standard supervision checklist in the national IDSR guidelines had not been completed by any of the respondents in the last six months before the survey despite reporting supervisory visits to the LGA and health facilities. The supervisory visits could not be substantiated with available reports in the state and LGA offices.

The major factors affecting the implementation of IDSR as identified by respondents at the state and LGA levels include the following:

- LGADSNOs limiting collection of IDSR data to only health facilities designated as focal sites for AFP surveillance of the polio eradication initiative
- inadequate IDSR reporting tools
- limited knowledge of the surveillance officers on case definitions and core indicators of surveillance
- limited capacity of the surveillance officers at the state and LGA levels to conduct supportive supervision
- limited capacities of the personnel to generate IDSR information products for decision making and public health actions
- lack of transportation facilities

A full overview of the factors impacting the implementation of IDSR can be found in Table 2.

**Table 2:** Summaries of the major factors affecting implementation of IDSR at the state and Local Government Areas levels in Northeastern Nigeria, 2017

Sites	Major observations
State levels	- IDSR Technical guidelines and standard case definitions were available in all offices but no evidence of usage by the staff - Very few private health facilities participated in IDSR implantation and reporting - No evidence of community based surveillance - No evidence of analysis of IDSR data other than on polio eradication initiative activities - All staff interviewed knew only timeliness and completeness as the core indicators for IDSR at both health facility and Local Government Areas levels - Supervision to the lower levels were done but there were no reports seen - The IDSR supervisory checklists were not used - There was no evidence of written feedbacks to the supervisees - Lack of motorbikes to facilitate supportive supervision
Local government levels	- IDSR technical guidelines and list of standard case definitions were available in the Local Government Areas offices but were not put to used. - DSNOs limit collection of IDSR data to only designated focal sites for AFP surveillance of the polio eradication initiatives - There was no evidence of recent analysis of IDSR data - There were supervisory visits to the lower levels but there were no reports of the activities. - Supervision were not done with the IDSR standard supervision checklists - Staff had been trained but there was no focus on basic concept of IDSR, identification, reporting, analysis and response to outbreak of diseases. - All the outpatient registers reviewed revealed missing data - Staff interviewed complained of lack of means of transportation for supervision and retrieval of IDSR data from the health facilities - The staff interviewed knew only timeliness and completeness as the core indicators for IDSR at the health facility level. - Lack of motorbikes for retrieval of surveillance data from the health facilities, supportive supervision, verification and response to outbreaks of diseases, conditions and events.

## Discussion

The rapid assessment of the status of implementation of IDSR in the three North East states in Nigeria revealed that the strategy was not functioning. The major gaps included number and types of health facilities involved in IDSR reporting; inadequate documentation of patients’ biodata and medical details in the health facility registers; and lack of personnel capacity to detect and report cases and collect, collate, analyze and interpret IDSR data. Other gaps included the inadequate capacity of key stakeholders to conduct supportive supervision; insufficient monitoring and evaluation of the system; and underuse of the tools for IDSR implementation and laboratory support networks for prompt confirmation of outbreaks of diseases, conditions and events of public health concerns.

### The distribution of health facilities reporting IDSR

The health care delivery system in Nigeria is a mixture of public and private health facilities. The private health facilities make substantial contribution in meeting the health needs of the populace in the country and are important foci for public health surveillance [16, 17]. The limited participation of health care facilities especially private health facilities in IDSR implementation in this survey indicates gaps in necessary surveillance data for the country. Similar findings on the participation of private health facilities in IDSR implementation were identified in Enugu where only 25% of staff in private health facilities, compared to 70% in public health facilities, were able to identify the correct forms for monthly IDSR reporting [10, 11, 18, 19]. With these gaps, diseases with potential for outbreak may go unnoticed until it is too late leading to increase in morbidity and mortality. Additionally, inadequate data used for decision making on planning and responses to public health events of concern may lead to an inadequate or inappropriate response. Involvement of all health facilities in IDSR will broaden the sphere of disease surveillance in the country and will produce more reliable data for decision making and planning for public health interventions. As such the government should ensure an all-inclusive approach for effective participation of the health facilities in the IDSR strategy.

### The source of IDSR data (health facility registers)

The quality and reliability of information generated for any surveillance system are only as good as the source of the data. Incomplete or missing data from source documents, as seen in the rapid assessment, will lead to misinformation and insufficient conclusions. For example, lack of laboratory results for most of the diseases treated at the health facilities means that they can only be classified as suspect cases and not confirmed. Additionally, as completing and extracting data from source documents to the IDSR forms is dependent on the type of registers, the process is overly complicated. For example, aggregating data from multiple registers with different designs is confusing and complicating, and the chance for error is high and there is a reduction in the quality and reliability of the information [11]. Harmonization of the multiple health facilities registers and trainings for clinicians on confirmation of disease using laboratory test by the relevant agency of government will ease the extraction of the data and improve its quality.

### IDSR reporting tools (forms)

The process of summarization of data from the health facility register to the IDSR reporting form is manual and paper-based. The LGA DSNOs are responsible for ensuring the availability of the reporting tools at the operational units. Unavailability of reporting tools will reduce the normal functioning of the IDSR strategy. The rapid assessment revealed that reporting forms were not readily available at the operational unit. The lack of form availability at the operational level could be due to virtual absence i.e. the LGA DSNO failed to distribute the commodity, or due to true absence i.e. because of failure of the state to produce. Similar findings were noted at the local government level in Yobe state, North East Nigeria where it was reported that unavailability of the reporting forms was a major challenge for the implementation of the IDSR strategy in the state. The study revealed that only 8% of the health facilities had the IDSR reporting forms [12]. Strengthening of the IDSR system in Nigeria must include the production and distribution of all reporting tools to all operational levels. The NCDC which is the country’s institution responsible for public health surveillance should ensure the production and distribution of the reporting tools to all levels of implementation.

### Capacities of the IDSR focal persons

The functionality of the surveillance system requires a chain of staff who have been adequately trained and are adequately supported. For example, if a clinician fails to document the right diagnosis or any of the variables in the patient treatment cards the recorder will not have the correct data available to input into the health facility register. Similarly, if a recorder fails to document the diagnosis of the patient in the health facility register extraction of the data into the IDSR reporting form will be in adequate and incorrect. The surveillance focal persons at the health facility level are responsible for detecting diseases for immediate reporting guided by the case definitions, completing the form for immediate reporting and transmitting the same to the LGA DSNOs for immediate actions. They are also responsible for the completion of the weekly and monthly forms. Extraction of the data from the register depends on the knowledge of the surveillance focal person on the national disease surveillance reporting system, case definition for each of the priority diseases, IDSR data management, production of IDSR information product for action, the IDSR forms and their uses and to whom to send the report [20–22]. A study in South East Nigeria, revealed that although the awareness of disease surveillance was as high as 90% among healthcare workers, only 33.3, 31.1, and 33.7% of them knew the specific uses of forms meant for immediate, weekly and monthly reporting respectively [23]. Their finding is similar to results of this rapid survey.

Training has been recognized as one of the veritable means to improve knowledge and a motivation for a positive attitudinal change of staff towards their work. Impact of training on staff knowledge on IDSR was confirmed by a study in Uganda, which found that increased staff knowledge on detection of IDSR priority diseases, reporting, data analysis and interpretation, preparedness and responses including increased staff confidence to perform IDSR tasks [24]. The benefits of IDSR training at the health facility level were also confirmed by a second study in Uganda where trainees cited some of the important benefits to include increased awareness and change in attitude about disease surveillance [25]. As important as training is in the improvement of knowledge and attitude, supportive supervision helps trainees to improve their performance. Supportive supervision is a key mechanism to ensure staff perform their tasks and activities according to set standards. It provides the opportunity for cordial interaction between the superior officer and their subordinate. When proper supervision is not in place it can negatively impact surveillance systems. Nyaaba et al. noted that poor supervision and lack of feedback to lower levels in the surveillance hierarchy as a major challenge for the disease surveillance system in Ghana [11].

### Adequate infrastructure

Adequate infrastructure is another necessary component in surveillance systems. The rapid assessment noted that office support equipment such as computers and printers for the surveillance teams both at the state and LGA levels were lacking in two of the three states surveyed. This lack of infrastructure negatively impacted basic data analysis, writing of reports and reproduction of forms. Access to adequate mobile technology will increase access to electronic surveillance which will be a more efficient and effective data collection method than the current paper-based IDSR reporting in Nigeria. Access to mobile technology will also overcome another current gap in transmitting information through paper forms. The simplest means of transportation for the state and LGA surveillance officers to the lower levels are motorbikes. However, due to the insurgency in the North East the government baned the use of motorbikes. This ban has negatively impacted the retrieval of IDSR data and the ability to perform supportive supervision [10, 11, 21]. It is obvious that paper-based reporting leads to serious limitations in the transmission of the data from the point of generation to the higher level (LGA). An electronic system deployed to the health facilities level is a better option to enhance timely reporting of the IDSR data for action. The government should explore the use of the electronic system for reporting of IDSR and develop the capacities of the focal persons on its usage to improve the system.

### Laboratory support for disease surveillance

The survey revealed limited utilization of a laboratory for confirmation of diseases prior to treatment at the health facilities. Symptomatic or empirical treatment leads to poor patient management and is a major cause of late detection and delay in outbreak reporting. This practice undermines the critical role of laboratory services to confirm suspected cases which is the backbone of the integrated diseases surveillance and response. Poor utilization of this critical element of the surveillance system was identified as one of the barriers for effective implementation of the IDSR strategy in Tanzania [10]. Furthermore, in response to disease outbreak, the laboratory provides the scientific evidence for the prevention and control of infectious diseases and no outbreak investigation and response is complete without the laboratory supports [26]. A well-functioning public health laboratory service for confirming suspected cases is the backbone of IDSR strategy. The government should ensure the availability of laboratories for the prompt confirmation of disease especially those with epidemic potential across the country.

### Limitations

The major limitation of this survey was the security issues prevalent in the states involved in the study. The security challenge influenced the selection of the study sites in each state as the insurgency was still active at the time of the survey. The survey team had to limit their visits to sites that were close to the state capital that are relatively safe and accessible. This could have influenced the results of the study because the facilities in the capital or close to the capital are more likely to benefit from supervision from the surveillance team.

## Conclusions

This rapid survey has revealed that IDSR was not implemented systematically in the three northeastern states of Nigeria. The major gaps were on the number and types of health facilities reporting IDSR, the documentations of patients’ data in health facility registers, the tools and logistics for IDSR reporting and the capacities of the surveillance officers to detect and report cases, collect, collate, analyze and interpret IDSR data and to conduct supportive supervision. A systematic approach to building the capacity of the surveillance officers on IDSR, provision of the data tools with support structures and strengthening of the public health laboratory services will improve the implementation and functionality of the strategy in Nigeria.

## Data Availability

The dataset used and analyzed during this study are available from the corresponding author on reasonable request.

## Abbreviations

AFP: Acute flaccid paralysis
DSNO: Disease surveillance and notification officer
FCT: Federal capital territory
IDSR: Integrated disease surveillance and response
LGA: Local government area
LGADSNO: Local government disease surveillance and notification officer
NCDC: Nigeria center for disease control
RDT: Rapid diagnostic test
RRT: Rapid response team
SDSNO: State disease surveillance and notification officer
WHO: World health organization
